# Tenofovir, Interferon Pathways, and Mucosal Immunity: Implications for People Living With HIV

**DOI:** 10.1111/aji.70255

**Published:** 2026-05-11

**Authors:** Florian Hladik, Sean M. Hughes, Claire N. Levy, Rachel L. Creighton, Romel Mackelprang, Germán G. Gornalusse

**Affiliations:** ^1^ Department of Obstetrics and Gynecology, Division of Research University of Washington Seattle Washington USA; ^2^ Department of Medicine, Division of Allergy and Infectious Diseases University of Washington Seattle Washington USA; ^3^ Vaccine and Infectious Disease Division, Fred Hutchinson Cancer Center Seattle Washington USA; ^4^ Department of Global Health University of Washington Seattle Washington USA; ^5^ Institute For Stem Cell & Regenerative Medicine University of Washington Seattle Washington USA

## Abstract

**Background:**

Antiretroviral therapy (ART) suppresses HIV replication and protects people living with HIV (PLWH) from progressing to AIDS. However, despite ART, many PLWH experience chronic immune activation, which contributes to premature aging and non‐AIDS‐related comorbidities. One reason for this chronic immune activation (CIA) is HIV and its reservoir. There is, however, accumulating evidence for an unexpected contributor: the nucleoside/nucleotide reverse transcriptase inhibitor (NRTI) class of drugs, particularly tenofovir, which can independently induce interferon (IFN) signaling in mucosal tissues. This CIA‐driving effect of tenofovir and other NRTIs warrants further study, as these therapies are taken lifelong and PLWH suffer disproportionately from inflammation‐related comorbidities.

**Objective:**

This review synthesizes evidence demonstrating that tenofovir contributes to CIA across multiple mucosal compartments (rectum, duodenum, and vagina) in both HIV‐uninfected and infected individuals. We detail the known mechanisms that likely underlie NRTI‐induced CIA: (1) Suppression of interleukin 10 (IL‐10), and induction of interleukin 12 (IL‐12) and interferon‐stimulated genes (ISGs), through inhibition of the protein kinase AKT, and (2) interferon response‐enhancing interactions with endogenous retroelements. Tenofovir also increases the number of specialized ISG^high^ epithelial cells in the gut. We further discuss the hypothesis that the chronic interferon signaling driven by NRTIs exacerbates HIV reservoir persistence and T‐cell exhaustion. Finally, we propose emerging therapeutic strategies, including NRTI‐sparing antiretroviral regimens and targeted immunomodulatory treatments, to reduce CIA and improve clinical outcomes for PLWH.

## 1 Chronic Immune Activation in People Living With HIV

### 1.1 Persistent Immune Activation Despite ART

Despite full suppression of viral replication by antiretroviral therapy (ART), chronic immune activation (CIA) and inflammation remain defining clinical features of HIV‐1 infection [1, 2, 3, 4, 5]. In both untreated and ART‐treated PLWH, biomarkers of immune activation are consistently elevated. These include increased levels of inflammatory cytokines such as interleukin‐6 (IL‐6), tumor necrosis factor‐α (TNF‐α), and interferon‐γ (IFN‐γ), as well as markers of cellular activation, including CD38 and HLA‐DR expression on T cells, and soluble markers such as sCD14, sCD163, C‐reactive protein (CRP), and D‐dimer [2, 6, 7, 8, 9]. Notably, even after virological suppression with ART, many PLWH experience insufficient CD4+ T cell recovery and incomplete immune reconstitution [10].

The persistence of immune activation despite viral suppression has profound clinical implications. CIA is associated with the accelerated onset of serious non‐AIDS events, including neurocognitive disorders, coronary artery disease, chronic liver and kidney dysfunction, metabolic syndrome, osteoporosis, and non‐HIV‐associated cancers [1, 2, 11]. This constellation of comorbidities reflects an accelerated aging phenotype, often termed “inflammaging,” in which PLWH experience age‐related conditions at younger chronological ages than the general population [12, 13, 14, 15], shortening their healthspan by an estimated 16 years [16, 17]. To set the stage in this section, we will briefly discuss possible causes of CIA in PLWH.

### 1.2 Putative Mechanisms of Chronic Immune Activation

#### 1.2.1 The HIV Reservoir and Residual Viral Production

The persistence of the HIV‐1 reservoir during ART contributes to CIA [18]. Latently infected cells harbor integrated proviral DNA [19]. These reservoir cells sporadically produce viral nucleic acids, proteins, and full particles, providing antigenic stimulation to the immune system. The HIV proteins p17, p24, and gp41 stimulate Toll‐like receptor (TLR)‐2 [20], and HIV RNA stimulates TLR‐7 and TLR‐9 [21]. Glycoprotein 120 binds to CCR5, inducing production of the inflammatory cytokine IL‐1β^22^. Reverse‐transcribed HIV‐1 DNA is recognized by IFI16, stimulating inflammatory cytokine production [23].

#### 1.2.2 Microbial Translocation and Gut Barrier Dysfunction

Microbial translocation across the gastrointestinal tract contributes to CIA during treatment [24]. HIV kills CCR5^+^CD4^+^ T cells in the gut [25], especially TH17 cells [26]. Their depletion, along with HIV‐induced inflammation, increases gut permeability, allowing microbes and their components, such as lipopolysaccharide (LPS), flagellin, and peptidoglycan, to enter the bloodstream, triggering immune activation and inflammation [27, 28, 29]. Elevated LPS levels are seen in chronically HIV‐infected individuals and do not fully normalize with ART [24]. HIV‐1 infection also alters the intestinal microbiota, a change that by itself associates with gut inflammation, microbial translocation, and increased peripheral T cell activation [30].

#### 1.2.3 Coinfections

Coinfection with other pathogens contributes to CIA in PLWH^2^. Despite ART, PLWH may experience more frequent reactivation of latent viruses such as cytomegalovirus (CMV), herpes simplex virus (HSV), and Epstein–Barr virus, which in turn drives CIA [31, 32, 33]. Women coinfected with hepatitis C virus (HCV) and HIV‐1 exhibit higher T cell activation compared to women living with HIV alone, and treatment of HCV reduces this T cell activation [34].

#### 1.2.4 Immune Dysregulation

Untreated HIV infection causes a variety of immune disruptions beyond the progressive depletion of CD4^+^ T lymphocytes. Many of these do not fully resolve with ART initiation, especially if ART is started long after primary infection. Regulatory T cells (Tregs), which are important to counterbalance immune activation [35], are depleted by HIV infection [36, 37, 38, 39, 40]. ART partially restores Treg numbers, but Tregs from PLWH retain elevated levels of inhibitory receptors, such as PD‐1 and CTLA‐4 [40]. T cell senescence, characterized by telomere shortening, upregulation of CD57, and downregulation of costimulatory molecules such as CD28 and CD27 [41], is accelerated by HIV infection and persists even with sustained virologic response to ART [42, 43]. Likewise, infection‐induced increases in the activity of the tryptophan (Trp)‐degrading enzyme indoleamine 2,3‐dioxygenase (IDO) may persist during ART, leading to the accumulation of the Trp metabolite kynurenine (Kyn). An elevated Kyn/Trp ratio is associated with inflammation and frailty (reviewed in [44]). HIV infection also causes fibrosis of lymphatic tissues, which is largely irreversible once established, contributing to immune dysfunction [45, 46].

#### 1.2.5 Genetic and Host Factors

Genetic predisposition influences the degree of immune activation during chronic HIV infection [47]. For example, polymorphisms in the IL‐10 gene have been associated with reduced mortality and slower CD4^+^ T cell loss in PLWH [48, 49]. Polymorphisms in the interferon regulatory factor 7 (IRF7) gene reduce IFN‐α responses of plasmacytoid dendritic cells to HIV‐1 [50]. Specific KIR‐HLA genotypes have been associated with failure to achieve immunological recovery despite ART that was effective in rendering plasma viral loads undetectable [51].

### 1.3 Sex Differences of Chronic Immune Activation

While the mechanisms described above apply broadly to PLWH, a large body of literature documents the influence of biological sex on immune responses (reviewed in [52]). Women generally mount more robust immune responses than men, with higher baseline levels of CD4^+^ T cells and stronger antibody responses. These differences may translate into distinct patterns of immune activation and clinical outcomes in HIV infection [53, 54]. Several studies have documented that women living with HIV on suppressive ART demonstrate higher levels of CIA compared to men [55, 56, 57]. These differences persist despite long‐term viral suppression, but their magnitude may vary across populations and with the specific CIA measurements used. For instance, one study found only marginal differences in immune activation between sexes under long‐term suppressive therapy [58]. Understanding sex‐specific immune differences among PLWH is important because immune activation may affect gynecological and pregnancy health.

### 1.4 Context for this Review

The mechanisms of CIA in PLWH listed above are well described in the literature. What has been relatively lacking is a consideration of the potential for antiretroviral drugs themselves to cause CIA. While ART dramatically reduces HIV replication and associated immune activation, accumulating evidence suggests that nucleoside/nucleotide reverse transcriptase inhibitors (NRTIs), such as tenofovir, may stimulate innate immune responses, particularly the type I/III interferon system, in mucosal tissues. The following section will examine the evidence for NRTI‐induced immune activation.

## 2 Immunological and Pro‐Inflammatory Effects of Tenofovir and Tenofovir‐Based PrEP in Clinical Studies

The antiretroviral drug tenofovir is a common component of drug combinations to treat PLWH and to prevent HIV infection [59, 60, 61, 62, 63, 64, 65, 66]. In particular, it is part of the most common oral pre‐exposure prophylaxis (PrEP) regimen, Truvada, which contains two NRTIs: tenofovir disoproxil fumarate (TDF) and emtricitabine (FTC). It has also been studied as a topical preventative agent for vaginal and rectal application. Its use as PrEP has allowed study of its effects on immune activation in the absence of HIV. Here, we describe the key features of tenofovir's immune‐activating effects in the mucosa: (1) inflammation and epithelial barrier disruption after topical administration to the mucosa; (2) induction of interferon‐stimulated genes at mucosal sites after both topical and oral administration; and (3) inhibition of the anti‐inflammatory cytokine IL‐10 and corresponding stimulation of pro‐inflammatory cytokines such as IL‐12.

### 2.1 Inflammation and Epithelial Barrier Disruption in the Vagina

Two studies of topical vaginal tenofovir administration suggest pro‐inflammatory and barrier‐disruptive effects on the vaginal mucosa. A Phase 1 trial of a TDF intravaginal ring revealed unexpected inflammatory responses. The trial compared a TDF ring to a placebo and was electively terminated early after 8 of 12 women (67%) assigned to the TDF ring developed Grade 1 vaginal ulcerations [67]. No ulcerations occurred in the placebo group. Multiple inflammatory cytokines and chemokines were significantly elevated at Days 14 and 28, with 5‐ to 15‐fold increases in IL‐1α, IL‐1β, TNF‐α, IL‐6, IL‐8, CXCL9, CXCL10, and other mediators. RNA sequencing of ectocervical tissue revealed an enrichment of inflammatory pathways, interferon responses, and T lymphocyte activation. Vaginal ulcers have also been described with the topical application of similar drugs: a clinical trial of 1% topical cream containing the nucleotide analog cidofovir reported mild‐to‐moderate ulcerations in 13 of 33 PLWH [68].

The precise mechanisms by which the TDF ring caused vaginal ulcers remain uncertain. The polymer itself was unlikely to be toxic, as the identical placebo ring caused no ulcerations. TDF is metabolized in vivo to tenofovir and formaldehyde, which could potentially contribute to topical irritation [69]. Toxic effects were not linked to vaginal microbiota changes, as no consistent microbiota alterations were observed [67]. Confusingly, in a prior study of the same intravaginal TDF ring in a similar number of participants, no ulcerations were observed [70]. However, that study tested the ring for only 14 days, whereas the ulcers arose after a mean of 32 days, indicating that mucosal damage accumulates over time and may not lead to clinically observable symptoms within 2 weeks of use.

Another notable difference was that the participants in the longer study were sexually active [67], whereas the prior study was conducted in sexually abstinent women [70]. Initiating vaginal sex has, by itself, been reported to increase cervicovaginal immune activation in adolescents and young adults [71], and microabrasions from sexual activity could enhance the drug's toxicity. Indeed, in vitro studies have shown impaired wound healing in primary genital epithelial cells and fibroblasts exposed to tenofovir or tenofovir alafenamide [72]. Notably, the use of this ring in six sexually inactive macaques for 6 months did not lead to ulcerations [73], also indicating that sexual activity could be a factor contributing to the toxicity of the intravaginal TDF ring. Alternatively, the number of animals may have been too small, or this preclinical animal model may simply not represent humans well.

The other study finding immune activation in the vagina following topical tenofovir application was MTN‐014. The study's unique crossover design allowed direct comparison of rectal versus vaginal application of 1% tenofovir gel in the same individuals over 14 days [59]. Tissue samples were collected from both vaginal and rectal mucosa at screening and after each product use period. Remarkably, rectal gel application affected not only rectal tissue but also induced the expression of similar gene sets in vaginal tissue [74], most notably the stimulation of type I/III interferon genes and the upregulation of gene sets related to epithelial‐mesenchymal transition (EMT) [74].

EMT is the process by which epithelial cells acquire motile and invasive characteristics consistent with mesenchymal cells. EMT can be involved in the generation of cancer stem cells and the resultant malignant disease processes [75]. While no publications exist for tenofovir, vaginal treatment with the NRTI azidothymidine in mice yielded a genital cancer rate of ∼25% [76]. EMT is also important for healing in human skin: re‐epithelialization and wound closure are correlated with the induction of EMT‐related genes in wound margin‐lining keratinocytes [77, 78]. No such data exist for vaginal wound healing, but the stratified squamous epithelium of the vagina is quite similar to the epidermis, except for its lack of superficial keratinization. Thus, speculatively, the induction of EMT processes by tenofovir could signify a response to vaginal barrier disruption rather than being a cause of it. However, certain types of EMT induction are associated with inflammation [78, 79], which could, in turn, compromise epithelial integrity.

### 2.2 Type I/III Interferon Pathway Stimulation: The Most Consistent and Persistent Mucosal Response

Induction of type I/III interferon (IFN‐I/III)‐related genes is the most consistent and persistent mucosal response to tenofovir, occurring with both oral and topical use, across multiple anatomical sites, and persisting for at least 2 months [74, 80].

Hughes et al. (2020) demonstrated that, compared to the pre‐treatment baseline in each individual, oral TDF/FTC induces IFN‐stimulated genes (ISGs) in rectal and duodenal biopsies from HIV‐uninfected individuals [80]. Thirteen genes were significantly upregulated in rectal tissue, with eleven being type I interferon pathway members: DDX60, HERC6, IFI6, IFI27, IFI27L1, IFIT1, ISG15, MX1, OAS1, RSAD2, and SAMD9. Induction of ISGs by Truvada was confirmed in two independent rectal cohorts (Spearman *r* = 0.91) as well as at two sites in the gut (rectum and duodenum, *r* = 0.81). Notably, TDF/FTC increased the number of ISG15‐bright epithelial cells in the gut (2.76‐fold in rectum, *p* = 0.0023; 1.37‐fold in duodenum, *p* = 0.06). Many of these cells co‐expressed GP2, a marker identifying microfold (M) cells, specialized intestinal epithelial cells with heightened immunological activity [81]. The increased frequency of these cells compared to pre‐treatment likely contributes to mucosal ISG production [80, 82].

The same ISGs induced by oral TDF/FTC were significantly enriched after 7, 14, and 56 days of daily rectal tenofovir gel use, with significance increasing with longer‐term use [74, 80]. Enrichment of the ISG gene set was also observed in the vagina after 14 days of daily topical use. Notably, ISG induction occurred not only with daily topical use but also with pericoital (event‐driven) rectal gel application, despite participants using the gel only 2.9 times per week on average.

Murata et al. (2018) documented significantly elevated serum levels of IFN‐λ3 (IL‐28B; a form of type III interferon) in patients treated with the nucleotide analogs tenofovir or adefovir (median 27.2 pg/mL) compared to those receiving the nucleoside analogs lamivudine or entecavir (median 1.4 pg/mL, *p* < 0.001) [83]. Table 1 lists all nucleotide and nucleoside analogs of historic and current clinical importance, but the authors tested only the four drugs mentioned. Follow‐up in vitro screens revealed that tenofovir induced IFN‐λ3 expression exclusively in colon‐derived epithelial cell lines, with no induction in hepatocytes, fibroblasts, lymphocytes, or peripheral blood mononuclear cells (PBMC). Others have shown that the IFN‐λ receptor (IFNLR1/IL‐10Rβ) is largely restricted to epithelial cells [84, 85]. This tissue tropism suggests that orally administered tenofovir induces IFN‐λ3 specifically in gut epithelial cells, from which it distributes systemically [83].

**TABLE 1:** **Clinically used nucleoside and nucleotide analog reverse transcriptase inhibitors**. Nucleosides must be phosphorylated three times inside the cell to become active; nucleotides only twice.

Drug	Abbreviation	Main clinical indication(s)
**Nucleoside analog RT inhibitors**		
Zidovudine	AZT, ZDV	HIV (obsolete)
Didanosine	ddI	HIV (obsolete)
Zalcitabine	ddC	HIV (obsolete)
Stavudine	d4T	HIV (obsolete)
Lamivudine	3TC	HIV, hepatitis B virus (HBV)
Emtricitabine	FTC	HIV, also HBV‑active
Abacavir	ABC	HIV
Telbivudine	LdT	HBV
Entecavir	ETV	HBV
**Nucleotide analog RT inhibitors**		
Tenofovir disoproxil fumarate	TDF	HIV, HBV
Tenofovir alafenamide	TAF	HIV, HBV
Adefovir dipivoxil	ADV	HBV (obsolete for HIV)

In summary, tenofovir, regardless of formulation, route, or combination with FTC, consistently activates mucosal interferon pathways, which could contribute to CIA.

### 2.3 Inhibition of the Anti‐Inflammatory Cytokine IL‐10 and Other Pro‐Inflammatory and Immunoregulatory Effects

In vitro investigations with human monocytic cell lines and PBMC have characterized tenofovir's effects on cytokine networks [86, 87, 88, 89]. Both Melchjorsen et al. (2011) and Murata et al. (2019) found that tenofovir strongly decreased IL‐10 production but increased IL‐12p70 levels, shifting the IL‐10/IL‐12 balance toward a more proinflammatory profile [86, 87]. This in vitro result was recapitulated in vivo following 7 days of daily tenofovir 1% gel application to the rectum in the MTN‐007 trial [90]. The gel strongly suppressed IL‐10 expression in the rectal mucosa, as well as the expression of two transcription factors involved in IL‐10 production, CREB1 and CREBBP [91]. This suppression of anti‐inflammatory pathways was accompanied by the induction of the chemokines CCL2, CCL19, CCL21, CXCL9, and CXCL13, and by increased levels of the lymphocyte markers CD2, CD3D, CD7, CD8A, and CD19. Immunohistochemical staining confirmed a 2‐ to 5‐fold increase in infiltrating T lymphocytes (CD3^+^ and CD7^+^) in the rectal mucosa [91]. Similarly, Biswas et al. demonstrated that in vitro treatment with tenofovir stimulates CXCL8 and CCL20 secretion by blood‐derived macrophages, dendritic cells, and activated CD4^+^ T cells [88]. To varying degrees, tenofovir also increased CXCL8, CCL20, and TNF‐α secretion from CD4^+^ T cells, macrophages, and epithelial cells isolated from the female reproductive tract [89].

Testing mouse cells in vitro, tenofovir and a panel of related nucleotide analogs stimulated proinflammatory mediators, including CCL5, CCL3, TNF‐α, IL‐1β, IL‐6, and nitric oxide from isolated hepatocytes, peritoneal macrophages, and spleen‐derived lymphocytes [92, 93, 94]. One in vivo mouse study, however, reported the opposite: a cocktail of four intraperitoneally delivered NRTIs, tenofovir, and the nucleoside analogs emtricitabine, zidovudine, and abacavir, diminished hepatic and systemic proinflammatory responses to bacterial infections, thereby preventing death by lethal LPS shock [95].

In vivo in humans, tenofovir has apparent immune‐activating effects in mucosal tissues [74, 80, 91], but studies in blood have been more contradictory [96, 97]. Castillo‐Mancilla et al. (2015) documented significant reductions in systemic T‐cell activation in healthy HIV‐negative adults after 30 days of Truvada, with activated (CD38/HLA‐DR coexpressing) CD8^+^ T cells decreasing from 3.97% to 2.71% (*p* = 0.03) and remaining suppressed at 2.41% thirty days after discontinuation [96]. In contrast, Richert‐Spuhler et al. (2019) observed divergent patterns in HSV‐2‐seropositive women on long‐term Truvada (24–36 months) [97]: peripheral blood showed increased activation markers, with elevated CCR5, CXCR4, CD38, Ki‐67, and PD‐1 on circulating T cells during PrEP; however, in the ectocervix, they observed reduced HIV target cell availability and immune quiescence, with significantly lower frequencies of CCR5^+^CD4^+^ target cells (4% during PrEP vs. 8% post‐PrEP, *p* = 0.0035), regulatory T cells, and CD68^+^ macrophages.

On the other hand, a recent study found that oral Truvada PrEP increased the serum concentrations of intestinal Fatty Acid Binding Protein (I‐FABP), a marker of epithelial injury [98]. I‐FABP levels were positively correlated with the clearance rate of a systemically delivered HIV‐1‐neutralizing monoclonal antibody, suggesting increased mucosal leakage. This insult to the gut mucosa could, over time, contribute to CIA and reduce the efficacy of concurrent antibody‐based immunotherapies.

### 2.4 Implications

In summary, topical and oral tenofovir have complex, duration‐dependent, and site‐specific effects on mucosal immunity. Stimulation of type I/III interferon pathways emerges as the dominant, persistent immunological signature of tenofovir, occurring across all formulations (oral TDF/FTC, topical gel), dosing regimens (daily, pericoital), mucosal sites (rectum, duodenum, vagina), and durations of use (7 to 56+ days).

Interferon pathway activation, combined with IL‐10 suppression and IL‐12 induction, is the defining immunological consequence of tenofovir/Truvada use. These effects occur specifically at mucosal surfaces, where HIV first invades [99, 100, 101], potentially contributing to antiviral efficacy by boosting innate immunity. However, persistent interferon stimulation over months to years could have detrimental consequences, including contributions to CIA, inflammaging, and increased non‐AIDS comorbidities in PLWH [74].

It is worth noting that most studies showing increased interferon expression were performed in HIV‐uninfected individuals, who likely have a lower level of background interferon expression compared to PLWH due to the absence of HIV. Future studies will be needed to determine whether tenofovir stimulates type I/III interferon pathways in PLWH. Furthermore, consistent immunological effects were only seen at mucosal sites directly exposed to tenofovir (duodenum and rectum after oral use, rectum and vagina after topical use). While these changes can, at least in part, be recapitulated in vitro using cell lines and PBMC, systemic effects in blood in vivo have been inconsistent, or, when observed, could be linked back to events in the exposed mucosa (IFN‐λ3, I‐FABP) [83, 98]. This suggests that systemic treatment may not cause similar effects at tissue sites not directly exposed to tenofovir, for example, the respiratory tract or the skin. Determining if secondary sites are affected will be important for gauging the anatomical extent to which the human body's immune system responds to tenofovir and other NRTI‐class drugs.

The known biological mechanisms underlying interferon induction by tenofovir will be discussed in the next section.

## 3 Potential Mechanisms by Which Tenofovir and NRTIs Modulate the Interferon System

**FIGURE 1:**
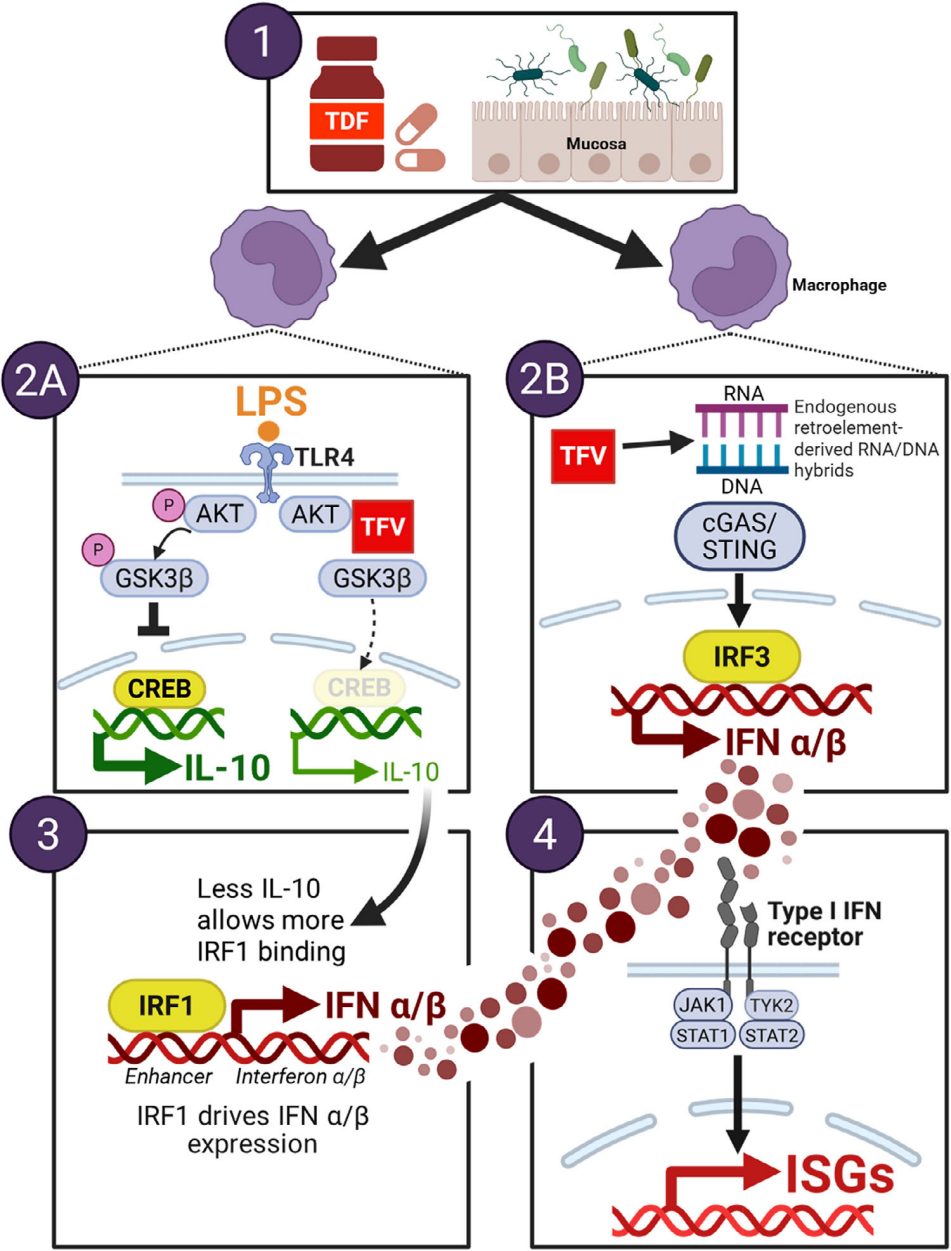
**Proposed model of tenofovir's contribution to chronic immune activation. Step 1: Cellular uptake and metabolism**. After oral administration, the diester prodrug tenofovir disoproxil fumarate (TDF) is absorbed through the gut mucosa and hydrolyzed by epithelial and plasma esterases to tenofovir (TFV). Circulating TFV is taken up by many cell types and phosphorylated to the active tenofovir diphosphate (TFV‐DP) by intracellular kinases. TFV‐DP terminates DNA synthesis during HIV reverse transcription because it lacks the 3’‐OH group required to form a phosphodiester bond with the next nucleotide. Monocytes/macrophages are the main producers of LPS‐induced IL‐10 [87, 102, 104], and we therefore postulate that tissue macrophages are key cell types mediating tenofovir's effects on innate immunity. **Step 2A:** **AKT inhibition and IL‐10 suppression**. When bacterial lipopolysaccharide (LPS), which is abundant in the gut lumen, binds to TLR4 on macrophages, the mammalian target of rapamycin (mTOR) pathway is activated. An early step in the mTOR pathway is the phosphorylation of the cytoplasmic serine/threonine kinase AKT, which, via intermediate steps (not shown), supports the downstream phosphorylation of the serine/threonine kinase GSK3β. GSK3β phosphorylation keeps it inactive in the cytoplasm, allowing nuclear IL‐10 transcription factors to bind and drive IL‐10 gene expression. This is depicted on the left side of panel 2A, with the cAMP response element‑binding protein (CREB) representing one of more than twenty known IL‐10 transcription factors [104, 105, 106]. The right side of the panel illustrates what occurs when TFV binds to AKT, blocking its phosphorylation and downstream mTOR signaling [87, 102]. As a result, GSK3β remains unphosphorylated and active, translocating to the nucleus and inhibiting IL‐10 transcription factor binding, thereby acting as a negative regulator of IL‐10 transcription. Of note, similar events may occur following stimulation with activators other than LPS, such as TNF‐α or live bacteria [86], but this has been less studied mechanistically. **Step 2B: Retroelement RT inhibition, cDNA:RNA hybrid accumulation, and innate immune sensing**. Step 2B has been demonstrated for the nucleoside analog lamivudine (3TC), but hypothetically should occur for tenofovir as well [103]. NRTIs act as chain terminators during reverse transcription (RT) of endogenous retroelements, including LINE‐1 retrotransposons and human endogenous retroviruses (HERVs) [107, 108, 109, 110]. In certain HERVs where RNase H polymerase activity is weak [111], DNA chain termination results in the cytoplasmic accumulation of truncated cDNA:RNA hybrids. These hybrids are recognized by pattern recognition receptors (cGAS, RIG‐I, DDX41), triggering activation of the interferon regulatory factor IRF3 [103, 112] and production of type I (IFN‐α, IFN‐β) and type III (IFN‐λ3; not shown) interferon [83, 102]. **Step 3: IL‐10 suppression and interferon induction**. Without tenofovir, IL‐10 suppresses LPS‐induced interferon expression by decreasing the chromatin accessibility of IRF1‐responsive genetic loci, which are essential for the transcription of type I IFN genes [113]. When IL‐10 is suppressed by tenofovir, the binding activity of IRF1 to enhancer regions of type I IFN genes is predicted to increase, enhancing IFN‐α/β production, which in turn stimulates ISG expression (Step 4). In macrophages, IRF1 has also been shown to enhance the expression of ISG genes directly, bypassing the need for upstream interferon production [114]. **Step 4: Synergistic activation of the interferon system**. Type I and III interferons, produced in parallel from Steps 2B and 3, bind to their receptors and broadly induce the expression of interferon‐stimulated genes.

The molecular mechanisms underlying tenofovir's immunomodulatory effects have only recently begun to be elucidated. Two key processes have emerged: (1) AKT kinase inhibition and mTOR pathway modulation, leading to strong suppression of IL‐10 [87, 102], and (2) inhibition of endogenous retroelement reverse transcription, leading to cytoplasmic accumulation of interferon pathway‐activating DNA:RNA hybrids [103]. Below, we describe these processes in more detail; a graphic scheme is depicted in Figure 1.

**FIGURE 2:**
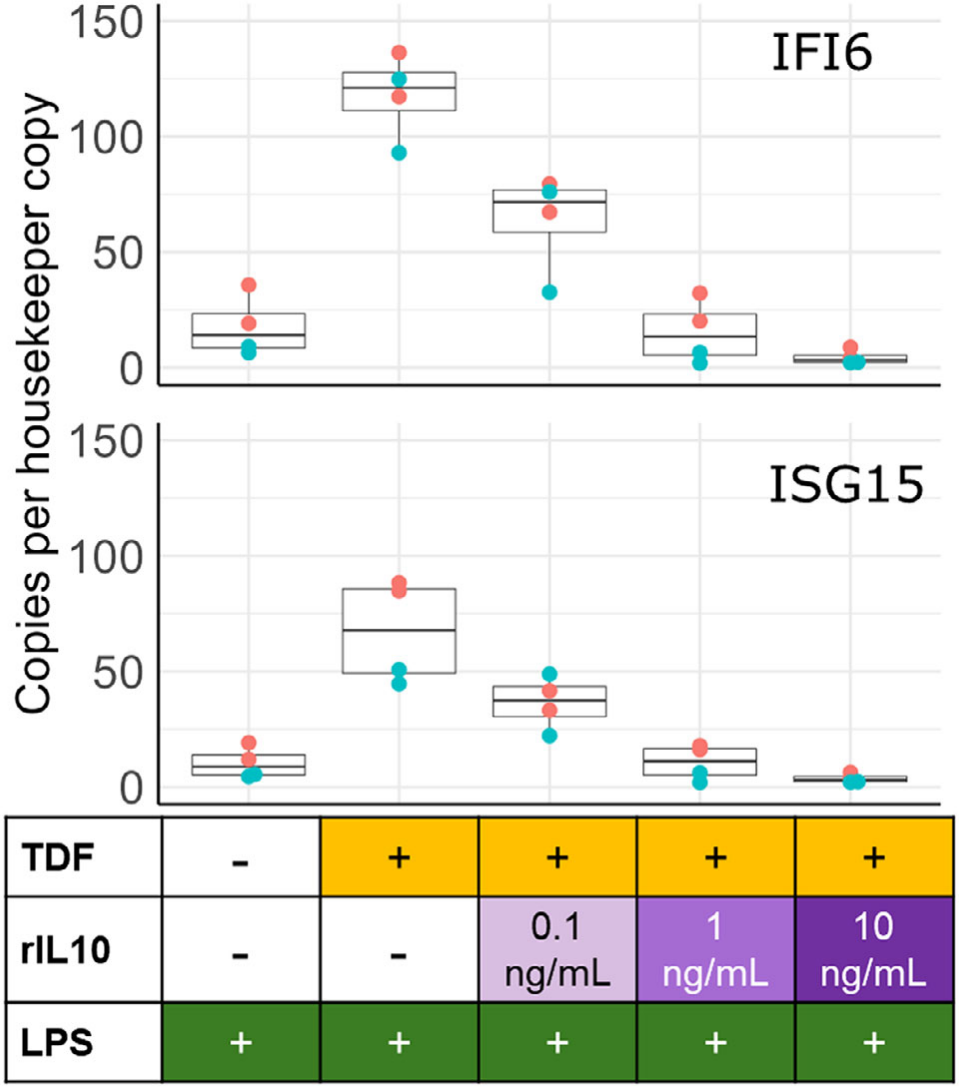
**An in vitro model of ISG enhancement by tenofovir and its reversal by recombinant IL‐10**. Adding TDF to LPS strongly induces ISG15 and IFI6 expression above the levels induced by LPS alone. IL‐10 reverses this effect in a dose‐dependent manner. TDF alone had no effect (not shown). Two blood donors (blue and red) were tested in independent experiments, and two cell culture replicates were included per condition. One million PBMC were used per condition and cultured in RPMI supplemented with 10% FBS, 1% penicillin/streptomycin, and 1% L­glutamine. Lipopolysaccharide (Invivogen Cat: tlrl‐eblps) was used at 100 ng/mL, and tenofovir disoproxil fumarate (TDF; NIH ARP Cat: 10198) at 25 µM. Recombinant IL10 (rIL10; Peprotech Cat: 200–10) was tested at 0.1, 1, and 10 ng/mL. The cells were cultured with TDF and IL‐10 for 2 h at 37°C and 5% CO_2_, followed by the addition of LPS for 24 h. After 26 h of culture, cells were lysed, and RNA was extracted. cDNA was prepared using the High‐Capacity cDNA Reverse Transcription Kit (ThermoFisher) and quantified by TaqMan assays for the interferon‐stimulated genes (ISGs) ISG15 and IFI6 on a Naica 6‐color Crystal Digital PCR instrument (Bio‐Rad). Boxplots show the median and the 75th and 25th percentiles. Whiskers extend to the lowest and highest values if they exceeded the 25th or 75th percentiles.

### 3.1 AKT Inhibition and mTOR Pathway Modulation (Step 2A)

The mechanism of tenofovir's IL‐10 suppression has been demonstrated in PBMC and monocytes in vitro. Rather than through MAPK or NF‐κB signaling [86], it occurs via binding to the serine/threonine kinase AKT [87, 102]. TFV binding prevents AKT translocation from the cytoplasm to the plasma membrane and its subsequent phosphorylation, thereby blocking phosphorylation of downstream effectors and the entire PI3K/AKT/mTOR signaling cascade [87, 102].

The functional consequences of AKT inhibition are profound for cytokine regulation, shifting the balance from anti‐inflammatory IL‐10 to pro‐inflammatory IL‐12p70. AKT phosphorylates and inactivates GSK3β, relieving its suppression of transcription factors required for IL‐10 expression. By preventing AKT phosphorylation, tenofovir maintains GSK3β in its active state, thereby suppressing IL‐10 transcription. Reduced IL‐10 production then reciprocally enhances IL‐12p70 and TNF‐α secretion through mechanisms that disinhibit pro‐inflammatory transcriptional programs [87, 102]. This shift in the IL‐10/IL‐12 balance towards IL‐12 favors Th1 responses and potentially viral clearance, but may contribute to CIA over the longer term.

Of note, this effect is NRTI subclass‐specific: only nucleotide analogs (tenofovir and adefovir) bind AKT, whereas nucleoside analogs (lamivudine, entecavir) lack this property due to the absence of the critical phosphonate moiety [83, 87, 102]. We predict that this difference leads to divergent immunological profiles in the clinic, which, if confirmed, could be a consideration for their use in HIV and non‐HIV contexts.

### 3.2 Interferon Regulatory Factor Activation Through IL‐10 Suppression (Step 3)

Recent insights from primary human monocytes may explain the connection between tenofovir‐mediated IL‐10 suppression and ISG activation. Mishra et al. (2025) demonstrated that IL‐10 inhibits inflammatory gene expression via interferon regulatory factor (IRF)‐class transcription factors, particularly IRF1 and IRF5, rather than via modulation of the canonical NF‐κB pathway [113]. IRF1 is a key factor in the response to LPS, as it is rapidly induced by TLR4 signaling and autocrine IFN‐β, and it drives transcription of type I IFN genes, ISGs, and inflammatory cytokines [113, 114]. IL‐10 interferes with this process, blocking IRF1's activity by reducing the acetylation of histone H3 lysine 27 (H3K27ac) at IRF1‐responsive genetic loci, decreasing their chromatin accessibility [113]. Thus, LPS induces ISGs via IRF1, and IL‐10 directly blocks this process. We postulate that by suppressing IL‐10, tenofovir allows IRF1‐mediated ISG induction to continue, resulting in higher ISG levels in response to LPS. Tenofovir‐mediated enhancement of LPS‐triggered ISG expression and its reversal by IL‐10 treatment can be readily observed in vitro (Figure 2).

### 3.3 Inhibition of Endogenous Retroelement Reverse Transcription (Step 2B)

A complementary mechanism of NRTI‐induced CIA posits that tenofovir inhibits reverse transcription of endogenous retroelements, leading to accumulation of immunogenic nucleic acid intermediates that trigger innate immune responses. Specifically, retroelements, including LINE‐1 (L1) and Alu retrotransposons, and certain human endogenous retroviruses (HERVs), such as HERV‐K, are transcribed from the human genome and then reverse‐transcribed. Both the nucleotide‐ and nucleoside‐analog NRTIs inhibit reverse transcription by terminating the growing nucleotide chain [107, 108, 109, 110], leading to cellular accumulation of immunostimulatory DNA:RNA hybrids. These hybrids activate ISGs via cytoplasmic nucleic acid sensors, including cGAS and RIG‐I/MDA5. Rajurkar et al. (2022) specifically demonstrated this mechanism for lamivudine (3TC) in colorectal cancer cells [103]. This mechanism is especially relevant in the colorectal mucosa, where retroelements are highly active [115], in part due to exposure to high concentrations of luminal LPS, which triggers retroelement transcription [116]. Additionally, L1 retrotransposons are constitutively expressed by the gastrointestinal epithelium [117]. Notably, the well‐described mitochondrial toxicity of NTRIs [118] leads to mitochondrial DNA (mDNA) release into the cytosol, adding another source of immunogenic nucleic acids [119].

Steps 2A and 2B likely happen synergistically, as the immunosuppressive activity of IL‐10 was recently discovered to function through epigenetic inhibition of ISG genetic loci (Step 3) [113]. This suggests that ISG induction in response to DNA:RNA hybrid accumulation is enhanced by the suppression of IL‐10.

### 3.4 Future Directions

Critical gaps remain in our understanding of how NRTIs contribute to CIA. Key questions requiring further investigation include: Do nucleotide and nucleoside analogs differ in their immunological profiles, given that both inhibit endogenous retroelement reverse transcription [107, 108, 109, 110], but only the former disrupt AKT/mTOR signaling? Another question is whether NRTIs may have countervailing effects. One group reported that NRTIs antagonize the ATP receptor P2X7, thereby blocking NLRP3 inflammasome activation and reducing immune stimulation [120, 121]. If confirmed, this would raise the possibility that NRTIs simultaneously exhibit pro‐ and anti‐inflammatory properties via the interferon and inflammasome axes, respectively. If so, what are the conditions favoring one or the other? Further, tenofovir's effects on HIV latent cells specifically are worth consideration. Olson et al. (2022) reported that the interferon pathway is disrupted in latently infected cells [122]. Would tenofovir overcome this disruption? Of interest is also whether polymorphisms in innate immune sensor genes (e.g. AKT, cGAS, TLR4, NLRP3) shape a person's susceptibility to NRTI‐induced immune activation. Lastly, we emphasize that while the described mechanisms add plausibility to the finding that tenofovir and other NRTI drugs stimulate the type I/III IFN pathway, it is crucial to confirm this in PLWH and to evaluate the extent to which this contributes to CIA. In fact, a recent report suggested that NRTI drugs may reduce age acceleration in PLWH [123]. Clearly, further studies are necessary to clarify the contributions of NRTIs and other ART drugs to CIA and immune aging, so that next‐generation combination regimens can be optimized for maximal antiviral efficacy while minimizing immunological perturbation.

## 4 Consequences of Chronic ISG Activation in People Living With HIV on Antiretroviral Therapy

PLWH experience an estimated 16‐year deficit in healthspan compared to people without HIV [16, 17]. This substantial quality‐of‐life deficit underscores that virological suppression alone does not normalize immune‐aging trajectories [124]. A key factor in this inflammaging phenotype may be chronic activation of type I/III interferon pathways. Understanding the long‐term effects of persistent interferon signaling is crucial for improving HIV treatment plans and addressing the paradox where drugs that reduce viral replication may unintentionally sustain CIA.

### 4.1 The Good and the Bad of Type I/III Interferons During HIV Infection

Type I interferons exhibit temporally divergent roles during HIV infection. During acute infection, robust IFN‐I responses inhibit viral replication, upregulate restriction factors, and initiate adaptive immunity. In fact, blocking IFN‐I signaling during acute SIV infection of rhesus macaques accelerates CD4 depletion and progression to AIDS, confirming IFN‐I's protective role early in pathogenesis [125]. However, persistent IFN‐I signaling during chronic infection transitions from beneficial to detrimental [126, 127]. The distinction between pathogenic and non‐pathogenic lentiviral infections in nonhuman primates illustrates this dichotomy: SIV infection of sooty mangabeys, characterized by a rapid resolution of the initially robust IFN‐I response, is nonprogressive despite continuing virus replication. In contrast, SIV infection in Asian rhesus macaques sustains elevated IFN‐I responses, which are thought to contribute to disease progression [128, 129].

In untreated PLWH, viremic individuals with low ISG expression tend to experience slower disease progression than those with high ISGs [130, 131, 132]. Moreover, elite controllers who maintain undetectable or extremely low viral loads without ART exhibit low ISG expression [132, 133]. This suggests that, over the longer term, ISG expression is both dispensable for viral control and deleterious to overall health.

PLWH on suppressive ART frequently retain elevated ISG signatures in gut mucosa, blood mononuclear cells, and tissues for years despite undetectable plasma viremia [58, 126]. As described in Section 2, oral TDF/FTC induces ISGs throughout the gastrointestinal tract even in HIV‐negative PrEP users, suggesting that tenofovir‐based ART might be one cause of persistently elevated ISGs in PLWH [80].

While there is yet little proof linking inflammaging in PLWH on suppressive ART specifically to the chronic activation of interferon pathways, persistent interferon stimulation is a well‐recognized mechanism of inflammaging [124, 134, 135, 136, 137]. Some direct evidence exists for HIV‐associated neurocognitive disorders, which affect 25%–50% of treated PLWH [138]. Tang et al. (2025) compared protein profiles of brains from PLWH on ART and people without HIV: the differences were largely immunological. 73.3% of elevated proteins in PLWH were immune‐related, and 23.3% specifically associated with IFN‐I signaling [139]. Single‐cell RNA sequencing of the brains revealed strong IFN‐I activity in astrocytes, microglia, and endothelial cells, even in the absence of detectable HIV. Similar mechanisms may also operate in other compartments.

Chronic IFN‐I exposure also drives progressive T‐cell exhaustion, characterized by upregulation of inhibitory receptors such as PD1 and proliferative senescence [140, 141]. While T cell exhaustion decreases the effector functions of the adaptive immune system, it also associates with low‐level CIA and inflammaging [142, 143, 144]. In HIV‐infected humanized mice, activation and exhaustion markers remained elevated on CD8^+^ and CD4^+^ T cells during chronic infection despite ART. IFNAR blockade reduced exhaustion markers, restored IFN‐γ and IL‐2 production upon HIV peptide stimulation, and decreased CD4^+^ T‐cell activation [126, 145, 146].

### 4.2 Chronic Interferon Signaling May Maintain HIV Reservoirs

Paradoxically, while type I IFNs initially control HIV replication, sustained IFN‐I signaling during ART may promote viral reservoir persistence by transcriptionally silencing integrated proviral DNA. Dickey et al. (2022) demonstrated in vitro that IFN‐I production in monocyte‐derived macrophages actively promotes latency by preventing NF‐κB p65 and RNA polymerase II recruitment to the HIV long terminal repeat (LTR) [147]. Blocking IFN‐I signaling prevented latency establishment, suggesting HIV exploits its own IFN‐I induction to create transcriptionally silent reservoirs. Thus, by inducing IFN production, tenofovir could help maintain the HIV reservoir.

There is further evidence that chronically high IFN signaling sustains the HIV reservoir. Using HIV‐infected humanized mice receiving tenofovir‐containing ART, Zhen et al. (2017) and Cheng et al. (2017) demonstrated that interferon‐alpha/beta receptor (IFNAR) blockade reduced the HIV reservoir compared to ART alone [145, 146]. Deeks (2017) previewed these findings, noting that “blocking the interferon response during ART may be a critical step toward eliminating the HIV reservoir.” [148]. Swainson et al. (2022) corroborated these results in SIV‐infected macaques: weekly anti‐IFN‐α antibody infusions during ART reduced lymph node viral DNA by 40% (*p* = 0.04) over 9 weeks [149]. In two human studies, however, IFN‐α treatment itself, alone [150] or in combination with a latency‐reversing agent [151], tended to reduce the latent HIV reservoir. The first study was a small pilot trial that reported a slight, statistically nonsignificant reduction in integrated DNA in peripheral blood CD4^+^ T cells over 20 weeks of IFN‐α treatment but lacked a randomized ART‐only arm [150]. The second study evaluated a single subcutaneous injection of IFN‐α, combined with three oral doses of the histone deacetylase inhibitor Panobinostat over 4 days, in PLWH on ART [151]. This short‐term induction of HIV reactivation and innate immune modulation skewed the HIV reservoir profile towards provirus integration in transcriptionally repressed heterochromatin regions. Follow‐up studies will need to determine the specific clinical benefit of IFN‐α in this combination.

These studies provide some support for the hypothesis that IFN‐induction by tenofovir during chronic HIV infection could unexpectedly increase the size of the HIV reservoir. Complementary mechanisms by which tenofovir‐based NRTIs may contribute to reservoir persistence have also been hypothesized. Hladik (2015) proposed that tenofovir may promote latency by: (1) directly driving cell proliferation, including cells harboring HIV provirus; (2) enhancing ISG activation and CIA through IL‐10 suppression, which further expands the clonal pool of latently infected cells; and (3) directly inhibiting transcription of integrated provirus [152], which decreases the visibility of reservoir cells to the immune system. Three reports suggested the last possibility by showing that NRTIs significantly decreased HIV reactivation from resting T cells in in vitro latency models [153, 154, 155]. Notably, however, the underlying mechanism remains unknown.

### 4.3 Tenofovir's Contribution: Compounding Chronic Interferon Activation?

As discussed above, chronic type I/III interferon signaling likely represents an underappreciated driver of HIV reservoir persistence, T‐cell exhaustion, neuroinflammation, accelerated aging, and excess morbidity in PLWH on ART. By stimulating type I/III interferon signaling, tenofovir‐based regimens may contribute to CIA [80, 83]. Thus, ART regimen optimization may be a strategy to ameliorate some side effects of CIA. This, and other mitigation strategies, will be discussed in Section 5.

## 5 Therapeutic Strategies for Reducing NRTI‐Associated Chronic Immune Activation

The accumulated evidence presented above suggests that tenofovir‐based NRTIs induce interferon signaling, potentially contributing to CIA as well as HIV reservoir persistence. These potential negative consequences motivate the investigation of interventions to reduce CIA by normalizing the overstimulation of the interferon system. Two complementary strategies merit clinical evaluation: (1) NRTI‐sparing antiretroviral regimens that eliminate the source of drug‐induced ISG activation, and (2) immunomodulatory therapies that block chronic interferon signaling or downstream inflammatory pathways. It is important to note that these strategies need to be investigated in clinical trials before being implemented in clinical practice. Indeed, the NIH Office of AIDS Research recently issued guidelines stating that changing the ART regimen or initiating immunomodulatory therapy solely to reduce CIA should only take place within clinical trials [156].

### 5.1 NRTI‐Sparing Antiretroviral Regimens

Many non‐NRTI‐class drugs, listed in Table 2, have been in use for many years, but need to be combined with NRTIs to achieve full viral suppression. Only the recent development of potent integrase strand transfer inhibitors (INSTIs) and novel long‐acting formulations [157] has enabled viral suppression without NRTI backbones. The oral NRTI‐sparing combinations of dolutegravir (DTG) with rilpivirine (RPV) and DTG plus ritonavir‐boosted darunavir demonstrated non‐inferiority to NRTI‐containing regimens in multiple randomized trials [158, 159, 160, 161, 162, 163]. Long‐acting cabotegravir plus RPV represents the first fully NRTI‐sparing injectable ART, administered intramuscularly monthly or bimonthly [164, 165, 166, 167, 168]. In addition, long‐acting lenacapavir, a first‐in‐class HIV‐1 capsid inhibitor [169, 170, 171, 172, 173, 174] is also being evaluated with bictegravir for first‐line NRTI‐sparing treatment [175, 176].

**TABLE 2:** Currently licensed, non‐NRTI‐class, antiretroviral drugs of clinical use.

Class	Drug
Non‐nucleoside reverse transcriptase inhibitors (NNRTIs)	Efavirenz, nevirapine, doravirine, etravirine, rilpivirine
Integrase strand transfer inhibitors (INSTIs)	Bictegravir, cabotegravir, dolutegravir, elvitegravir, raltegravir
Protease inhibitors (PIs)	Lopinavir, ritonavir, atazanavir, darunavir
Entry inhibitors – CCR5 antagonist	Maraviroc
Entry inhibitors – fusion inhibitor	Enfuvirtide
Post‐attachment inhibitors	Ibalizumab
Capsid inhibitors	Lenacapavir

The advantages of the new NRTI‐sparing regimens in terms of limiting CIA still need careful evaluation. Clinical trials should compare NRTI‐sparing regimens and tenofovir‐containing ART in virologically suppressed PLWH with elevated inflammatory markers, measuring gut mucosal ISG expression [80], plasma IFN‐λ3 [83], T‐cell exhaustion markers [145, 146], inflammatory biomarkers (IL‐6, CRP, D‐dimer, I‐FABP [8, 9, 98, 177]), and proviral reservoir size [178, 179] over at least 6 months. Ideally, this should be done using a prospective, randomized, crossover study design switching PLWH from NRTI‐containing to NRTI‐sparing regimens, so that each participant serves as their own control.

### 5.2 Immunomodulatory Adjuncts to Antiretroviral Therapy

Given that chronic interferon signaling and the unbalancing of IL‐10/IL‐12 likely contribute to CIA and reservoir persistence, targeted interferon and related pathway antagonists represent rational therapeutic adjuncts. Several biologics and small molecules warrant investigation.

Anifrolumab, a fully human monoclonal antibody blocking IFNAR1, demonstrated acceptable safety in lupus trials [180, 181]. Ruxolitinib, a JAK1/2 inhibitor approved for myelofibrosis, polycythemia vera, and graft‐versus‐host disease [182, 183, 184], blocks signaling downstream of IFNAR, IL‐6R, and other cytokine receptors. Both drugs could be tested to antagonize chronic ISG induction in PLWH. Indeed, in a first‐in‐class randomized clinical trial, ruxolitinib significantly decreased inflammatory markers in PLWH [185].

The mTOR inhibitor sirolimus (rapamycin) and the calcineurin inhibitor tacrolimus, which modulate T‐cell signaling and metabolism, are used in transplantation [186, 187, 188]. Small pilot studies in PLWH showed that sirolimus reduced T‐cell activation, increased central memory CD4^+^ T cells, and limited IFN‐I‐mediated inflammation, though clinical benefits remain uncertain [189, 190, 191, 192, 193].

IL‐10 pathway modulation presents another opportunity. A recent study revealed that IL‐10 directly antagonizes type I interferon responses [113]. Pegylated IL‐10 derivatives (pegilodecakin), tested in cancer immunotherapy, could theoretically dampen IFN‐I‐driven inflammation [194], but effects on HIV reservoirs remain untested.

Ustekinumab, an IL‐12/IL‐23 antibody approved for psoriasis and Crohn's disease [195], could antagonize the enhancement of LPS‐induced IL‐12 induction by tenofovir [87]. However, no data yet link IL‐12/23 blockade to HIV outcomes. In fact, during early HIV infection, higher plasma IL‐12 levels were associated with lower viral loads and improved maintenance of CD4^+^ T cell counts [196]. This finding could make IL‐12 blockade problematic for PLWH.

Hydroxychloroquine inhibits TLR9 stimulation and thereby mechanistically inhibits TLR‐mediated interferon induction and ISG expression [197]. Rother et al. (2020) demonstrated that hydroxychloroquine reduces interferon responses to viral stimuli and IFNs themselves. One study already reported that hydroxychloroquine reduces immune activation in PLWH [198].

Lactobacilli‐derived β‐carboline compounds represent a novel microbiome‐based immunomodulatory strategy with potential relevance to both HIV prevention and treatment. Glick et al. (2024) identified that vaginal *Lactobacillus crispatus* produces a family of β‐carboline alkaloids with potent anti‐inflammatory activity [199]. The lead compound, perlolyrine, suppresses both TLR‐mediated NF‐κB and type I interferon signaling in primary human monocytes. Intriguingly, topical vaginal application of perlolyrine significantly reduced genital inflammation in a mouse HSV‐2 infection model without compromising viral control in epithelial cells [199]. This cell‐type‐specific immunomodulation, suppressing pro‐inflammatory TLR/IFNAR signaling in immune cells while preserving epithelial antiviral defenses, could address a key limitation of indiscriminate interferon blockade. Orally or systemically administered β‐carboline derivatives such as perlolyrine could suppress chronic IFNAR/TLR signaling in PLWH on ART, potentially reducing reservoir persistence and inflammaging.

The above immunomodulatory treatments could be tested to antagonize persistent ISG stimulation and CIA in PLWH, with measurements including reservoir decay, immune reconstitution (CD4:CD8 ratio normalization), and reductions in inflammatory markers. However, these drugs should be carefully monitored for immunosuppressive effects, especially when used long‐term. Immunosuppression could lead to complications such as opportunistic infections, herpes zoster reactivation, exacerbation of CMV or HBV infections, and enhanced risk of active tuberculosis. To limit side effects, such immunomodulatory drugs could be tested as a short‐term pulse to reset the interferon system to a lower level of activation. This strategy, although without a clear precedent, could be effective when paired with a simultaneous reduction in interferon pathway stimulation by switching to an NRTI‐sparing ART regimen. Some drugs may also be less prone to causing immunosuppression than others. For example, IL‐10 derivatives are being designed to retain the desirable properties of IL‐10, such as opposing interferon system activation, while eliminating undesirable ones, including general immunosuppression [200]. Natural compounds such as β‐carboline derivatives, which are present in a healthy vagina, may also act as anti‐inflammatories without causing dangerous immunosuppression.

### 5.3 The Impact of Biological Sex and Other Host Factors

It is critical to account for sex‐specific variation in immune responses [52]. Differences in sex hormones [201, 202], immunity in reproductive tissues [203], and gene expression due to the X and Y chromosomes (such as X‐linked TLR7 escape [204]) cause differences in immune responses between sexes. The menstrual cycle [205], sexual activity [71], pregnancy, contraception, menopause, and hormone therapy [202] can all modulate immunity. This includes gender‐affirming hormone therapy. For example, testosterone treatment during PrEP with FTC/TAF decreased cervical FTC‐TP and TFV‐DP levels [206], which likely affects the drug's immunomodulatory activity as well.

The vaginal microbiome is a unique ecosystem that influences mucosal immunity in ways distinct from the gut, skin, or the penile environment [207, 208, 209]. The recent discovery that *L. crispatus* produces anti‐inflammatory β‐carbolines that suppress type I interferon signaling highlights this fact [199]. Bacterial vaginosis (BV) may modulate the immune effects of tenofovir in the female reproductive tract because BV‐associated bacteria metabolize the drug [208]. Pre‐existing genital inflammation, in response to BV or sexually transmitted infections, has also been shown to influence the local activity of antiretroviral drugs [210, 211]. Excellent reviews have been published on the relationship between the microbiome and the bioavailability of antiretroviral drugs [212, 213].

Other host factors worth consideration are the genetic determinants of drug bioavailability. For example, polymorphisms in genes encoding tissue‐specific kinases that catalyze the conversion of tenofovir to its active form, tenofovir diphosphate, can compromise drug activity [214], thereby likely modulating its CIA‐inducing effects as well. Genetic variants in drug transporters such as OAT1 may also influence tenofovir's activity [215], and may do so differently across tissues [216]. Similar principles apply to many other drugs, whose efficacy and toxicity are influenced by interindividual genetic variation in drug‐metabolizing enzymes and transporters [217].

Strategies to mitigate persistent activation of the interferon system and CIA in PLWH should consider that many factors, including those described in this section, may influence their effectiveness.

## 6 Conclusion

In conclusion, tenofovir‐based ART induces chronic interferon signaling, which may contribute to CIA, accelerated aging, and perpetuation of HIV reservoirs. This finding opens new opportunities for comorbidity reduction and could potentially be relevant for HIV cure research. NRTI‐sparing regimens combined with adjunctive immunomodulators (including microbiome‐based β‐carboline compounds) may represent a new frontier extending beyond viral suppression toward immune restoration. The convergence of mechanistic insights from mucosal immunology, pharmacology, microbiome science, and interferon research enables the design of rigorous trials to test these hypotheses and improve outcomes for people living with HIV.

## Funding

The author(s) declare that financial support was received for the research and/or publication of this article. This work was supported by the following grants from the National Institutes of Health: R01 AI172111, R01 AI184122, R01 AI116292, R01 AI134293, R21 AI174903, KL2 TR002317, and P30 AI027757. The funders had no role in study design, data collection and analysis, decision to publish, or preparation of the manuscript.

## Conflicts of Interest

The authors declare no conflicts of interest.

## Generative AI Statement

The authors used Perplexity, an AI assistant powered by GPT‑5.1, to support literature searches and summarize select prior work, and Grammarly, an AI‑assisted language‐editing tool, to improve spelling, grammar, and readability. All AI‑generated content and suggestions were reviewed, edited, and verified for accuracy by all authors, who take full responsibility for the final manuscript.
